# Water use and conservation in the operating room and perioperative setting: A scoping review

**DOI:** 10.1371/journal.pone.0341144

**Published:** 2026-01-30

**Authors:** Nicholas Kramer, Jay Zhu, Maya Xolal Herzig, Duncan Alexander Meiklejohn

**Affiliations:** 1 Department of Otolaryngology-Head and Neck Surgery, Henry Ford Macomb Hospital, Clinton Township, Michigan, United States of America; 2 Department of Surgery, University of New Mexico Health Sciences Center, Albuquerque, New Mexico, United States of America; 3 Division of Otolaryngology-Head and Neck Surgery, Department of Surgery, University of New Mexico Health Sciences Center, Albuquerque, New Mexico, United States of America; National Research and Innovation Agency, INDONESIA

## Abstract

Public health and food security in the United States depend on a reliable supply of fresh water. Freshwater availability is worsening, and prolonged drought conditions have affected large portions of the country. Hospitals are major consumers of water, and processes related to the operating room may present an opportunity to improve water conservation. A scoping review of the MEDLINE, Science Citation Index-Expanded, Emerging Sources Citation Index, and Embase databases was conducted for articles addressing water use and interventions for water conservation from January 1, 1948, to March 26, 2025. The review identified twenty-six studies meeting inclusion criteria, addressing four subsets of operating room and perioperative processes. The preponderance of published studies addressed water use during surgical hand scrubbing, with the remainder evaluating water use related to textiles, equipment, or sterilization processes. Interventions supported by high-quality evidence for reducing water use included turning off idling sterilizer equipment when not in use. Moderate to low quality evidence favored converting to waterless scrub techniques and/or on-demand scrub sinks, reusable alternatives for equipment, and non-cotton reusable textiles. Our findings identify several focused areas with potential for improving water conservation in the operative and perioperative setting. Further research is needed to comprehensively evaluate operating room, perioperative and overall hospital processes for water use, and to describe effective interventions for conservation.

## Introduction

Public health in the United States (US) depends heavily on fresh water. Over 30 million Americans now live in areas of high water limitation, and water availability is worsening [1–3], particularly in the context of the current Southwestern US megadrought [4–6]. Water scarcity also compromises food security, and millions of acres of US farmland no longer can support industrial-scale agriculture due to loss of aquifer water [7,8]. The geographical impact and severity of drought are expected to increase secondary to climate change, worsening this scarcity [9].

Hospitals are major consumers of water, with large buildings accounting for ~340 million liters (L) annually [10], and operating room (OR) processes are known to exert an outsized environmental impact within hospitals [11]. The scientific literature on water conservation related to the OR and perioperative processes is sparse and represents an area of opportunity for water conservation.

This scoping review investigates and categorizes the existing literature on water use in the OR and perioperative setting with the aim to 1) identify and evaluate the most impactful interventions for water conservation and 2) identify gaps within the existing literature. These results provide insight to healthcare professionals, administrators, and public health experts to guide current water conservation efforts and future research.

## Materials and methods

### Search strategy

The Preferred Reporting Items for Systematic Reviews and Meta-Analyses extension for Scoping Reviews (PRISMA-ScR) checklist was followed; the protocol was not pre-registered. A library professional assisted in designing the search and protocol for article inclusion. The research was exempt from institutional ethics review as it only assessed previously published studies and did not involve human or animal subjects or field research. The MEDLINE, Science Citation Index-Expanded, Emerging Sources Citation Index, and Embase databases were searched for the following terms from January 1, 1948, to March 26, 2025:

1) exp water/ or exp water purification/ or wastewater/ or (wastewater or water)
2) Operating rooms/ or exp surgical equipment/ or ((operating or surgical) adj3 (room* or theat* or suite* or equipment))
3) exp waste management/ or exp environment, controlled/ or greenhouse effect/ or exp conservation of natural resources/ or exp sterilization/ or (conserv* or recycl* or green or environ* or consum* or sustainab* or waste or sterilize* or disinfect* or ventilat* or heating or climate or resource*).ti,ab. or (air adj3 condition*)

Final search combined 1 and 2 and 3.

### Inclusion/exclusion criteria

Peer-reviewed and gray literature articles were reviewed; gray literature and multiple databases were included to attempt to capture all possible studies meeting inclusion criteria. Inclusion criteria included: 1) reports of original data, 2) quantification of water use and/or interventions conserving water in the hospital setting, 3) studies modeling hospital water use. Exclusion criteria included articles lacking original data, opinion pieces, non-medical reports, dental studies, lay press reporting. Outcomes included quantification of water use and quantification of water conserved through interventions.

### Screening, extraction, and synthesis

Title/abstract review was conducted independently by NK/DAM. Four independent reviewers (NK, MH, JZ, DAM) completed full text review, data abstraction, and analysis, with extracted data documented in a spreadsheet. Variables extracted included study design, topic, methods, key findings/outcomes, and quality rating. Inter-rater reliability was ensured via strict adherence to inclusion/exclusion criteria, independent review of all articles followed by discussion to clarify details and confirm a shared mental model, and a requirement for unanimous consensus on all decisions for inclusion/exclusion and rating. Disagreements were resolved with live group discussion of all articles and ratings, with unanimous consensus reached in all cases.

### Rating system

The quality of the articles was initially assessed using a standard framework for medical research publications, and overall quality was found to be low; only two small-scale handwashing studies [12,13] could be categorized as well-designed controlled trials without randomization, and the remainder were of lower quality. However, many articles using high-quality methods appropriate for environmental impact studies (e.g., life cycle assessment) did not fall into traditional quality rating categories for medical research studies, leading to a lower rating. This was felt to not accurately represent the quality of the studies. To address this issue, the evaluation framework established by Rizan and colleagues [14] in their investigation of carbon emissions within the operating room was adapted to assess water use literature. The modified system assesses the articles based on four key domains: completeness, consistency, transparency, and accuracy, with additional subcategories in the domains of transparency and accuracy. Each category or subcategory is scored between 0 and 2, reflecting an increasing alignment with each respective category. Ultimately, each article is assigned a percentile score out of a maximum score of 20, or a maximum score of 18 for noncomparative studies (a study was categorized as noncomparative if it did not perform a measured comparison of outcomes between two alternatives or compare measurements before and after intervention).

An example of modification to adapt this system to our study is as follows: The Consistency subdomain 3b in the Rizan system reads “To what extent are the greenhouse gases included clearly stated? *Number of GHGs included clearly stated (2) Number of GHGs deducible (1) Number of GHGs not deducible (0)*”. This subdomain was modified in our system to “To what extent is the quantity of water measured clearly stated? *Quantity of water included clearly stated (2) Quantity of water deducible (1) Quantity of water not deducible (0)*”. For this subdomain, an article which reported exact quantities of water would receive a score of 2; an article in which the quantity was deducible from graphic data or could be calculated from reported results would receive a 1, and an article in which the exact quantity could not be determined would receive a zero.

All included articles were reviewed and rated independently by NK, JZ, and DAM and disagreement was resolved with discussion and consensus. Studies were divided into three quality tiers: low (score ≤50%), moderate (51–75%), and high quality (76–100%). See S2 Table in the Supporting Information for full detail of all rating system categories as well as rating system results.

## Results

**Fig 1:**
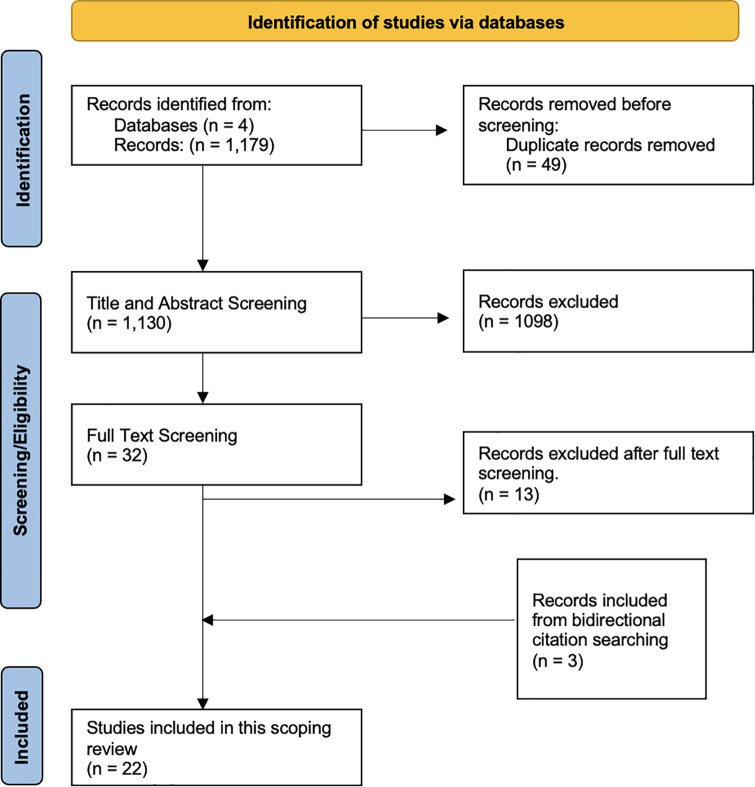
PRISMA diagram of articles included in review.

Systematic review identified 2099 articles, and 54 duplicates removed, leaving 2045**. B**ased on title/abstract review, 1953 articles were excluded. Full text review of 92 articles excluded and additional 69 articles. Bidirectional citation searching identified three additional articles which were included after full text review. Final review, data extraction and rating were performed on 26 articles meeting inclusion criteria. Article processing is depicted in Fig 1. Included articles are summarized in Table 1.

**Table 1:** Articles included in review.

Study/Year	Country	Category	Methodology	Data source(s)	Water outcomes
Ahmed 2007 [17]	Nigeria	Surgical hand scrubbing	Prospective observational audit	126 observations of 63 total staff members	20.2L used per scrub session
Carannante 2024 [23]	Italy	Surgical hand scrubbing	Quantitative observational audit	15 total surgical cases, exact number of observations not reported	20L used per water-based scrub, 0.006L used per alcohol-based scrub session
Gasson 2023 [18]	Wales (U.K.)	Surgical hand scrubbing	Quantitative observational audit	3 total observations	20.6L used per 3-minute water-based scrub, 6.9L used per one-minute scrub
Javitt 2020 [15]	U.S.	Surgical hand scrubbing	Quantitative observational audit	3 observations each of 8 scrub sinks	15.9L used per scrub session
Jehle 2008 [16]	England (U.K.)	Surgical hand scrubbing	Quantitative observational audit	30 observations of scrub sessions	18.5L used per scrub session
Jones 2009 [21]	Australia	Surgical hand scrubbing	Quantitative observational audit	3 observations of each of seven scrub techniques	Range 6.7-24.9L used per scrub session
Kara 2021 [19]	Turkey	Surgical hand scrubbing	Quantitative observational audit	Number of observations not reported	2.9L used (motion sensor) vs. 22.5L (continuous flow)
Petterwood and Shridhar 2009 [12]	Australia	Surgical hand scrubbing	Quantitative observational audit	10 observations each of two scrub techniques	4.5L used (flow only when soaping/ rinsing) vs. 15.5L (continuous flow)
Potgieter 2020 [13]	South Africa	Surgical hand scrubbing	Nonrandomized controlled before-and-after study	32 participants, 12–18 each scrub technique, number of trials not reported	Range 0.8L used (alcohol) to 5.7L (water scrub, no intervention)
Restaino 2024 [22]	Italy	Surgical hand scrubbing	Quantitative observational audit	Number of trials not reported	Range 0L used (alcohol) to 8.2L (povidone-iodine)
Somner 2008 [20]	Scotland (U.K.)	Surgical hand scrubbing	Quantitative observational audit	25 observations each of two scrub techniques	10.7L per water-based scrub with elbow-activated tap, 5L per scrub with leg-activated tap
Westwood 2023 [24]	U.K.	Surgical hand scrubbing	Prospective controlled nonrandomized study	23 observations each pre- and post-intervention	Education increased waterless scrub use from 15% to 17% of cases, 111L water saved overall with education intervention
Wormer 2013 [25]	U.S.	Surgical hand scrubbing	Quantitative observational audit	100 observations	Education increased waterless scrub use from 22% to 80% of cases
Burguburu 2022 [29]	France	Textiles	LCA, water use as outcome measure	LCA data from product manufacturers and published literature ^a^	Disposable option uses 95% less than reusable
Cohen 2023 [28]	Netherlands	Textiles	LCA, water use as outcome measure	LCA data from product manufacturers ^a^	Reusable options use 50–80% less than disposable
Vozzola 2020 [27]	U.S.	Textiles	LCA, water use as outcome measure	LCA data from direct measurement and public utility data ^a^	Reusable option uses 69–83% less than disposable
Adler 2004 [33]	Germany	Sterilization	Measurement of reprocessing water	3 test runs of reprocessing equipment	Processing one equipment load uses 434L
McGain 2014 [30]	Australia	Sterilization	Prospective comparative cohort study	305 circuits tested, 100 + each experimental arm	Cleaning equipment every 24h, 48h, or 7d was equivalent for sterility
McGain 2016 [31]	Australia	Sterilization	Retrospective quantitative audit	8760 hours of data from four unique sterilizers	1/5 of water use occurs during idle/standby mode
McGain, Moore 2017 [32]	Australia	Sterilization	Prospective quantitative audit	Meter monitoring of sterilizer every 5 min for a year	58L/kg of equipment sterilized, 5400L/d overall/sterilizer
Bertolo 2024 [39]	Italy	Equipment	Comparative cost analysis	Cost analysis	60L/procedure reusable, zero with disposable
Boucheron 2022 [38]	France	Equipment	Waste audit with water measurement	Direct measurement, cost analysis	60L/procedure reusable, zero with disposable
Eckelman 2012 [35]	U.S.	Equipment	LCA, water use as outcome measure	LCA data from direct measurement and product manufacturers ^a^	Reusable option uses 60% less than disposable
McGain 2010 [36]	Australia	Equipment	LCA, water use as outcome measure	LCA data from product manufacturers ^a^	Reusable option uses 70–90% less than disposable
McGain, Story 2017 [37]	Australia	Equipment	LCA, water use as outcome measure, various scenarios tested	LCA data from direct measurement and product manufacturers ^a^	Disposable options use 4–62.7% less than reusable
Rouviere 2023 [34]	France	Equipment	LCA, water use as outcome measure	LCA data from direct measurement and product manufacturers ^a^	Reusable option uses 10L less per intubation than disposable

L = liters, LCA = life cycle assessment, LMA = laryngeal mask airway, min = minutes, sec = seconds, U.K. = United Kingdom, h = hours, d = days, kg = kilograms.

^a^In LCA, local site-specific data inputs may be generated from direct measurement; multiple data points may not be routinely collected. Most data incorporated in LCA are not directly measured but obtained from published life cycle inventories/databases, where they are calculated as a weighted average from multiple sites and data sources.

Included studies were assigned to one of four intervention categories: Surgical scrubbing, Textiles, Sterilization, and Equipment. Using the modified rating system, 30.7% (8/26) of all included studies were of low quality, 57.7% (15/26) were of moderate quality, and 11.5% (3/26) were of high quality (S2 Table in Supporting Information).

### Surgical scrubbing

Thirteen articles examined water use related to surgical scrubbing. These were divided into three sub-categories: 1) baseline water use, 2) “on demand” vs. constant-flow water taps, and 3) alcohol-based waterless scrubs compared to water-based techniques.

#### Baseline water use.

Four studies quantified baseline water use related to surgical scrubbing. Javitt et al. found that a single 2-minute scrub session consumes 15.9L water [15], while Jehle et al. found that a 3-minute scrub session consumed 18.5L water with a sink flow rate of 6.2L/min. The authors estimated 60.2L water used per procedure based on an average of 3.3 scrubbed persons [16]. Ahmed measured average total scrub time as 4.8 minutes with a sink flow rate of 4.2L/min using 20.2L water, with active scrubbing accounting for only 1.4 minutes or 5.9L [17]. Gasson et al. found an initial three-minute scrub utilized 20.6L water at a sink flow rate of 6.9L/min. The authors estimated switching to alcohol-based scrub to replace the one-minute scrubs could save 82.3 - 288.7L per OR per day at their institution [18].

#### “On demand” vs. constant flow water taps.

Four studies evaluated “on demand” scrub sink taps, which allow cessation of water flow when not actively needed. Kara et al. reported that motion sensor taps ran for an average of 23 seconds and used 2.9L water during a three-minute scrub, compared to 22.5L consumed using a tap turned on continuously throughout the scrub. The authors estimate motion sensor taps would save 58.8L per surgical procedure assuming three individuals scrubbed [19]. Petterwood and Shridhar measured water use with continuous flow throughout the scrub session versus running water only when soaping and rinsing, and demonstrated that limiting active water flow only to when needed saved 11L water per scrub session compared to constant flow [12]. Somner et al. observed scrubs using either leg- or elbow-activated taps and found that leg-activated taps used 5.7L less water per scrub session (savings of 53%) compared to the continuous flow pattern of the elbow-activated taps [20]. Jones evaluated various permutations of elbow vs foot pedal scrub sinks, combined with low vs high tap flow rates for total water use. Lowest water use per scrub was 6.7L (foot pedal control, low flow rate, intermittent pattern of use) and highest was 24.9L (elbow lever control, high flow rate, continuous pattern of use) [21].

#### *Alcohol scrub* *vs. water-based* *scrub.*

Five studies compared alcohol-based hand rub sterilization to water-based scrubbing. Potgieter et al. evaluated three water-saving interventions: an initial water scrub followed by alcohol-based scrubs; an assistant limiting tap water flow to only that necessary for scrubbing; or encouraging staff to manually stop the water themselves when not actively scrubbing. The baseline scenario (hand washer choice of washing technique) used 5.7L water per scrub, alcohol scrub 0.8L, staff-assisted scrub 2.3L, and taps controlled by the hand washer used 1.9L [13]. Restaino et al. compared povidone-iodine, chlorhexidine-gluconate and alcohol-based scrub agents and determined alcohol-based agents to use the least water per scrub (0L), with chlorhexidine more (6.1L) and povidone-iodine the most (8.2L). The authors estimate 68,967L water/year could be saved with use of an alcohol-based scrub [22]. Carannante et al calculated water consumption of 20L per 5-minute water-based scrub, compared to 6mL consumed of alcohol-based scrub agent per scrub. The authors estimated 60L water consumed per procedure with water-based scrub versus 0.02L per procedure with alcohol-based scrub, with yearly water and cost savings of 71,476L and €1584 ($1867) at their institution with use of alcohol-based scrub [23].

Two studies compared waterless to water-based scrub and also evaluated interventions to increase use of waterless scrub techniques. Westwood et al. measured water use during scrubbing and reported average water consumption using water and soap scrub technique of 18.5L/scrub session. They then tested an educational intervention encouraging use of alcohol-based scrubs. The authors found water use was modestly decreased by 111L in a 3-week post-intervention observation period, with an increase in alcohol-based scrub use from 15% to 17% of eligible cases [24]. Wormer et al. identified that 98% of OR staff left water running continuously during scrubbing. Implementation of a hospital water savings campaign increased alcohol-based scrub use from 22% of scrub episodes to 80%. Water consumption per scrub session was not reported, but the authors projected possible annual water savings of roughly 2.7millionL with conversion to waterless scrub [25].

### Textiles

Three articles evaluated water use related to OR textiles, using life cycle assessment (LCA). LCA comprehensively quantifies environmental impacts and costs across all life cycle stages of a process, product, or system, and is well established for application in medical systems [26].

Vozzola et al. compared synthetic reusable to synthetic disposable surgical gowns for their consumption of fresh water. The study found that for every 1,000 uses, reusable gowns consumed 185L versus 1,097L for disposable gowns [27]. Cohen et al. compared the water usage of synthetic reusable to synthetic disposable OR head covers over the course of one year at a single tertiary care hospital. The authors found that reusable polyester (PE) head covers consumed 80% less water than disposable viscose and 50% less than disposable polyethylene options (32,000L for disposable viscose head covers, 13,000L for disposable polypropylene, and 6,000L for reusable) [28]. Burguburu et al. found that reusable scrub suits composed of 35% cotton and 65% PE consumed 95% more water than disposable suits made of 100% PE (6070L per four years of use by one staff member, compared to 300L for disposable), largely attributed to the substantial water required to produce the cotton for the reusable suits [29].

### Sterilization

Four articles evaluated water use related to sterilization and washing of instruments. McGain et al. evaluated bacterial contamination of anesthesia breathing circuits by rinsing the circuits after use and plating the resulting fluid on bacterial growth plates to quantify colony-forming units. Various time intervals between cleaning of the circuits were evaluated for their impact on bacterial contamination. The authors found no difference in bacterial colony-forming units if the time interval of circuit cleaning was increased from every 24 hours to every 7 days. Annual water savings were projected at 47,900L per year in a 300-bed hospital with use of the longer interval [30]. Two additional studies by McGain et al. examined the water consumption of equipment sterilizers and found that sterilizers consume 81/L water/hour during idling/standby mode, including a warm-up cycle whenever a sterilizer is idle for 2 or more hours. Overall, a fifth of sterilizer water was consumed during idling/standby mode, and heavier sterilizer loads were found to be more water-efficient. Strategies to selectively turn off a single hospital’s sterilizers could reduce annual water use by 450,000–1 million L [31,32]. Adler et al. tabulated water used in washing instruments used for laparoscopic cholecystectomy, and projected water used for this purpose at nearly 375,000L over five years [33].

### Equipment

Six articles evaluated water use related to equipment used in surgical procedures. Four studies used LCA methodology: Rouviere and colleagues found that reusable laryngoscope blades save 10L water per intubation compared to single-use disposable blades, or 221,600L/year at their institution based on 17,200 intubations/year [34]. Eckelman and colleagues compared water consumption between reusable and disposable laryngeal mask airways (LMA) and determined that reusable LMAs use 60% less water than disposable LMAs [35]. McGain et al. reported fully reusable anesthetic drug trays consume 3.1L water per use, compared to 10.3L for a disposable tray, and 26.7L for a disposable tray with cotton gauze pads and a paper towel added. The authors project savings of 70,000–100,000L water annually with use of reusable tray [36]. A second study led by McGain evaluated water consumption of reusable versus disposable anesthesia equipment, including breathing circuits, face masks, LMAs, and laryngoscopes. Several scenarios were evaluated; overall the authors found increased annual water consumption with increasing the proportion of reusable equipment, ranging from 30,900L/year (almost all disposable equipment) to 82,200L/year (all reusable equipment) [37].

Two studies which did not employ LCA focused on cystoscopes and compared water consumed for a single cystoscopy procedure using reusable equipment vs. disposable equipment. Both reported identical findings of 60L of water per procedure consumed by reusable cystoscopes and 0L water for the disposable cystoscope [38,39].

## Discussion

Existing research on water conservation methods in the operative and perioperative setting were collected. Only 26 total studies were identified; most were based in either Europe or Australia, and five were from the same lead author (Table 1). Notably, despite including peer-reviewed literature starting in 1948, the earliest report meeting inclusion criteria was published in 2004. The scope of topics is severely limited: only four subject areas were identified, and half (13/26) focused only on surgical scrubbing. There was wide heterogeneity in study design, scope, and data quality. Environmental studies often do not fit into typical frameworks for medical research publications; however, under the modified rating system for environmental studies only three studies reached the threshold of high quality (S2 Table in Supporting Information). Overall, the literature supports some basic interventions for water conservation. However, more comprehensive and quantitative studies are required to better understand overall OR water use in order to stratify the processes and procedures that consume the most water and identify the areas for greatest improvement. This review identified only one pair of studies by the same author that employed water meters to directly quantify real-life water consumption [31,32]. Limitations of this review include the absence of an established framework for assessing quality of environmental studies within the medical literature; this was addressed by adapting a previously published framework.

### Surgical hand scrubbing

Methodology varied across the evaluated surgical hand scrubbing studies, and time spent scrubbing and measured amounts of water used during scrub sessions varied significantly. All studies making direct, measured comparisons between handwashing techniques were limited by short duration, small numbers of study participants and non-blinding, and lack of rigorous technique for measuring water used. Most studies also failed to repeat measurements across multiple sinks and OR settings; these limitations may over- or underestimate water use.

Crucially, no published studies used LCA to comprehensively evaluate the water consumption from manufacture of waterless scrub products, however there is one LCA-based study currently in the data analysis stage, which compared chlorhexidine gluconate water-based scrub to a chlorhexidine gluconate/ethyl alcohol-based waterless scrub. Preliminary findings from this work confirm that water use is significantly higher in water-based scrub compared to waterless scrub, even when accounting for production and transport, including each product’s packaging (A. Artsen, personal communication).

Despite the variability of study design and other limitations noted above, all studies in this review found waterless scrub techniques superior for water conservation, and “on-demand” taps superior when water-based scrub technique is used. Importantly, these methods are equivalent in antisepsis efficacy for surgical procedures after an initial water-based scrub [40,41]. For future studies, LCA methodology is recommended to compare water-based to waterless scrub technique (including tabulating water consumed in manufacture of product containers, packaging, and disposable sponges/brushes), use of sink flow meters for direct measurement rather than estimation, that studies include increased numbers of observations and study participants, and expansion to more scrub settings. There also is a need for further quality improvement studies, with specific outcome measures, on interventions to promote water-friendly scrub techniques.

### Textiles

Whether reusable or disposable textiles are favored depends upon the textile composition; plastic polymer-based reusable textiles are water-saving compared to disposable textiles [27,28] while cotton materials were found to consume significantly more water due to agricultural water requirements [29]. Though more water-intensive, reusable cotton textiles are superior in seven out of ten overall environmental impact categories, including greenhouse gas release and particulate pollution [29]. A growing literature also emphasizes the health risks of plastic products, including but not limited to harms from tissue accumulation of micro- and nano-plastics [42,43] and adverse effects on a broad spectrum of human organ systems [44]. It is possible that reusable organic-based textiles may ultimately be preferred from a public health standpoint despite their higher water footprint. Future studies should incorporate LCA or similar comprehensive methodology to quantify the evaluate the various health implications of textile options.

### Sterilization

Optimizing sterilization efficiency is a promising target for reducing water consumption. Reducing equipment idle time has previously been shown to have potential in improving efficiency and cost savings [45–47] and moderate- to high-quality research by McGain et al. in this review confirms water savings as well [31,32]. A third study by the same group addressed decreasing the frequency of anesthesia circuit cleaning, with resulting projected water and cost savings [30]. In this study bacterial counts only and not actual infections were reported, however the authors point out that the rate of ventilator-associated infection in outpatient surgery is so low as to make this a less meaningful indicator, and that prior studies also support the safety of decreased cleaning frequency [48–50]. Future research in this area should focus on repeating these findings in other countries and settings and comparing the water consumption of high-level disinfection and waterless sterilization/disinfection techniques.

### Equipment

Studies addressing use of disposable versus reusable equipment options were mixed. Studies by Boucheron and Bertolo found disposable cystoscopes significantly superior to reusable, however neither addresses the water required for manufacture of the disposable instruments. The production of raw plastic alone consumes a significant amount of water, ranging from ~15-66L/kg plastic [51,52], in addition to the previously noted adverse health effects of plastics. Manufacturing processes for a complex surgical instrument such as a cystoscope, requiring multiple plastic and non-plastic materials, are expected to consume still more, multiplied by the number of procedures performed. Indeed, three studies that accounted for the water consumed by disposable equipment manufacture found that the reusable option was clearly superior for water consumption [34–36]. Finally, McGain and colleagues found that water use was significantly increased with reusable anesthesia equipment compared to disposable, however their scenario involved a hospital in Australia with an electrical power mix relying heavily on polluting, non-renewable energy generation, including coal [37]. Coal-based electricity generation consumes large quantities of water compared to renewable energy sources such as photovoltaic or wind [53]; it is expected that a cleaner energy mix powering hospital disinfection and sterilization would reduce the water consumption of the reusable options. Overall, reusable options for surgical and anesthesia equipment appear to be favored for water use in studies employing rigorous methodology; however, this effect is likely variable and may be setting dependent. Future studies should account for all life cycle stages for both reusable and disposable equipment options.

## Conclusions

Overall assessment and research recommendations: High-quality research is lacking to guide water conservation in the operative, perioperative and hospital settings, and water consumption in most hospital and OR processes remains undescribed. Based on this review, weak recommendations for water-conserving interventions include converting to waterless scrub techniques and on-demand scrub sinks, turning off idling sterilizer equipment, and favoring non-cotton reusable textiles and reusable alternatives for equipment. Future research should prioritize the use of water meter data and longitudinal accounting of water usage to more accurately quantify water consumption, focusing on a larger breadth of operating room processes. The importance of higher quality measurements is highlighted by the large proportion of hand-washing studies identified in this review, none of which attached a water meter to a scrub sink to quantify and compare true water usage over time. Furthermore, this review identified no studies examining water usage during surgery or during OR turnover and sanitation. These areas for future research involve local operating room processes where both baseline water consumption and conservation outcomes can be quantified through rigorous accounting. For processes such as equipment manufacturing and waste disposal that extend beyond hospital operations, life cycle assessment (LCA) remains the gold standard for quantifying environmental impact. Overall, this review highlights the need for more rigorous accounting of water utilization in all operating room processes in order to measure and guide water conservation efforts.

Practical recommendations: Recommendations for policymakers and hospital administrators based on the results of this study include: 1) optimizing steam sterilizer equipment use schedules to minimize water-wasting idle time, 2) mandating and encouraging use of waterless surgical scrub for OR personnel, 3) changing constant-flow sinks for surgical hand scrubbing to motion-activated or other “on-demand” style of faucet, 4) preferring reusable surgical equipment over disposable alternatives, 5) incorporating reusable energy sources for hospital processes whenever possible, and 6) purchasing and using reusable synthetic textiles when the alternative is a disposable synthetic product. Reusable cotton-based textiles consume more water than synthetic alternatives, however the water-saving benefit of synthetic textiles when compared to cotton may be offset by the adverse environmental and health effects of synthetic materials, particularly for disposable products.

## Supporting information

S1 Table PRISMA Checklist. (DOCX)

S2 Table Quality assessment of included studies. 1° = primary, 2° = secondary, CI = confidence interval. * Denotes a noncomparative study.
(XLSX)
